# A Set of Novel Microsatellite Markers Developed for a Distylous Species *Luculia gratissima* (Rubiaceae)

**DOI:** 10.3390/ijms12106743

**Published:** 2011-10-12

**Authors:** Wei Zhou, Dezhu Li, Hong Wang

**Affiliations:** 1 Key Laboratory of Biodiversity and Biogeography, Kunming Institute of Botany, Chinese Academy of Sciences, Kunming 650201, China; E-Mails: zhouwei@mail.kib.ac.cn (W.Z.); dzl@mail.kib.ac.cn (D.L.); 2 Graduate School of the Chinese Academy of Sciences, Beijing 100049, China; 3 Plant Germplasm and Genomics Center, Germplasm Bank of Wild Species, Kunming Institute of Botany, Chinese Academy of Sciences, Kunming 650201, China

**Keywords:** *Luculia gratissima*, distylous, microsatellite marker, polymorphism, population genetics

## Abstract

*Luculia gratissima* (Wall.) Sweet (Rubiaceae) is a perennial shrub distributed in the southeast margin of the Tibetan Plateau in southwest China and adjacent region of Nepal and Myanmar. The plant is a distylous species with reciprocally placed stigmas and anthers in each floral morph. By using the Fast Isolation by Amplified Fragment Length Polymorphism (AFLP) of Sequences Containing (FIASCO) repeats protocol, 19 primer sets were identified in two wild populations. Of these primers, 10 displayed polymorphisms and nine were monomorphic. The number of alleles per locus ranged from two to five, values for observed and expected heterozygosities ranged from 0.000 to 1.000 and from 0.289 to 0.760, with averages of 0.303 and 0.555, respectively. These microsatellite loci will facilitate further studies on breeding system, gene flow patterns, and population structure of *L. gratissima* and its allied species.

## 1. Introduction

*Luculia gratissima* (Wall.) Sweet (Rubiaceae) is an evergreen perennial shrub distributed in the southeast margin of the Tibetan Plateau in southwest China and adjacent region of Nepal and Myanmar at altitudes between 800 and 2400 m [1,2]. The species flowers from August to December, and produces several compact pink to white tubular flowers on each tight inflorescence that are sweetly fragrant. It is very interesting that the species is distylous with complementary positioning of stigmas and anthers in the two floral forms [3]. Previous studies suggest that the floral syndrome of distylous species would facilitate disassortative mating during pollinator visitation [4,5]. However, the potential functions of distyly are not well understood, and DNA microsatellites would provide a precise molecular marker to trace the characteristic of pollination patterns by parentage analysis. In this study, we have developed and characterized 19 microsatellite markers for *L. gratissima* using the Fast Isolation by Amplified Fragment Length Polymorphism (AFLP) of sequences containing repeats (FIASCO) [6], which will be used for further studies of breeding system, gene flow patterns, and population structure.

## 2. Results and Discussion

A total of 275 positive clones were captured, among these 102 clones (37%) were found to contain simple sequence repeats (SSR). Finally, 33 sequences contained SSR loci were selected for primer design. Nineteen microsatellite loci successfully amplified in *L. gratissima* for 33 microsatellite loci and 10 of them were polymorphic amplification, the remaining nine microsatellite loci were monomorphic as a result of capillary gel (Table 1).

The number of alleles ranged from two to five in 24 individuals of the species sampled from the two natural populations. Values for *H*_O_ and *H*_E_ ranged from 0.000 to 1.000 and from 0.289 to 0.760, with averages of 0.303 (SD = 0.285) and 0.555 (SD = 0.133), respectively (Table 2). For all 10 microsatellite loci, except LG2 and LG25, the genotypic frequencies showed significant deviation from Hardy-Weinberg equilibrium (HWE) (*P* < 0.01) indicating the possibility of null alleles and the disassortative mating of this distylous species.

These microsatellite markers developed in our study will be a useful tool for further studies of population genetics, and will be used to assign parentage to seeds which will help us understand the characteristic of pollination patterns for this distylous plant.

## 3. Experimental Section

Genomic DNA samples of *L. gratissima* were extracted from silica-gel-dried leaves of three different individuals using a modified hexadecyltrimethylammonium bromide (CTAB) method [7]. The extracted DNA was dissolved in 30 μL TE buffer. The fast isolation by AFLP of sequences containing repeats (FIASCO) [6] was performed in this study. Total genomic DNA (approximate 250–500 ng) was completely digested with 2.5 U of *Mse* I restriction enzyme (New England Biolabs, Beverly, MA, USA), and then ligated to an *Mse* I AFLP adaptor (5′-TAC TCA GGA CTC AT-3′/5′-GAC GAT GAG TCC TGA G-3′) using T4 DNA ligase (Fermentas, Burlington, ON, Canada). The digested-ligated fragments were diluted in a ratio of 1:10, and 5 μL of them were used amplification reaction with adaptor-specific primers (5′-GAT GAG TCC TGA GTA AN-3′/5′-TTA CTC AGG ACT CAT CN-3′). The amplified DNA fragments (200–800 bp) were enriched by magnetic bead selection with a 5-biotinylated (AG)_15_, (AAG)_10_ and (AC)_15_ probe, respectively [6]. The Recovered DNA fragments were reamplified with *Mse* I-N primers. The purified PCR products using EZNA Gel Extraction Kit (Omega Bio-Tek, Guangzhou, China), were ligated into pBS-T II vector (Tiangen, Beijing, China), and then transformed into *E. coli* strain DH5α competent cells (TaKaRa, Dalian, Liaoning, China). The positive clones were picked out and tested using vector primers T3/T7 and primer (AC)_10_/(AG)_10_/(AAG)_7_ respectively to select appropriate fragments which contained SSR. In other words, a set of tested PCR included three reactions was performed using T3 and T7, T3 and (AC)_10_, (AC)_10_ and T7 as primers, respectively. The second set of tested PCRs was done using T3 and T7, T3 and (AG)_10_, (AG)_10_ and T7 as primers, respectively. The last set of tested PCRs was done using T3 and T7, T3 and (AAG)_7_, (AAG)_7_ and T7 as primers, respectively. All these PCR reactions had the same conditions: 95 °C for 3 min followed by 30 cycles at 94 °C for 45 s, 52 °C for 1 min, 72 °C for 1 min, and a final extension step at 72 °C for 7 min. The positive clones were captured for sequencing with an ABI PRISM 3730XL DNA sequencer (Applied Biosystems, Foster City, CA, USA). Sequences contained simple sequence repeat and enough flanking regions were selected for primer design using Primer Premier 5.0 program [8].

The designed Primer pairs were assessed in 24 individuals of *L. gratissima* pooled from two natural populations collected in Nepal: NMD (Daman, Makwanpur, Narayani: 27°40′N, 85°05′E, 1970 m a.s.l.) and BLG (Godawari, Lalitpur, Bagmati: 27°40′N, 85°19′E, 1385 m a.s.l.). Herbarium voucher deposited in Kunming Institute of Botany, Chinese Academy of Science (code ZW0153-0176). The PCR reactions were performed in 20 μL of reaction volume containing 10–50 ng genomic DNA, 0.5 μM of each primer, 10 μL 2× Taq PCR MasterMix (Tiangen; 0.1 U Taq Polymerase/μL, 0.5 mM dNTP each, 20 mM Tris-HCl (pH 8.3), 100 mM KCl, 3 mM MgCl_2_). PCR amplifications were conducted under the following conditions: 95 °C for 3 min followed by 32–35 cycles at 94 °C for 30 s, at the annealing temperature for each specific primer (optimized for each locus, Table 1) for 45 s, 72 °C for 1 min, and a final extension step at 72 °C for 7 min. The amplification products were separated and visualized using QIAxcel of capillary gel electrophoresis system (QIAGEN, Irvine, CA, USA).

The data was analyzed by GENEPOP 4.0 [9], which included test of observed heterozygosity (*H*_O_), expected heterozygosity (*H*_E_), and departure from Hardy-Weinberg equilibrium (HWE) for the 10 polymorphic microsatellite loci.

## 4. Conclusions

In summary, 19 microsatellite markers have been specifically developed for *L. gratissima* in this study. The high discriminatory power of 10 polymorphic loci suggests that they should be suitable for survey of population structure and parentage analysis in this distylous species. These developed and characterized SSR markers for *L. gratissima* would also be useful for exploring genetic diversity and genetic structure of other species in *Luculia*.

## Figures and Tables

**Table 1:** Primer sequences and characteristics of 19 microsatellite loci successfully amplified in *Luculia gratissima*.

Locus	Primer sequence (5′–3′)	Repeat motif	Size range (bp)	*T*_a_ (°C)	GenBank Accession No.
LG2 *	F: ATGCTACACTTTCATCTCGGTA R: CGGTTGGAAGCTAAAATG	(GA)_14_	255–271	57	JN625264
LG5 *	F: GTAGGGTAAGAGTGGGTTG R: GTTTGGGAGTGGTTTGAT	(CT)_10_- (CA)_14_	166–170	60	JN625265
LG6	F: TCTTGGTCCTTTACTGGC R: TCATGCGAAATTCTCCAC	(CA)_12_	214	60	JN625266
LG10 *	F: GAAGCCCATTCCTGTTAC R: AAGCATTAGGCAAAGTCA	(GAA)_4_- (CA)_11_	153–165	61	JN625267
LG11 *	F: TAGAAACATACCCACCTG R: ACACTTCCAGAAAACCTC	(GAA)_7_	280–295	61	JN625268
LG12	F: ATCATTCAGGCTGACACG R: CAAATCCCAATACTTTCG	(GT)_8_ (GA)_11_	189	54	JN625269
LG13 *	F: CTACTTCTTGATCCTTCT R: TAGCATGTTGTAAATGTC	(CT)_6_ (CA)_10_	132–138	55	JN625270
LG14	F: AAAAAAGAAGACGAGAGCA R: GCCGCAGATGTAAATAGG	(AG)_15_	219	60	JN625271
LG15	F: CCAAAGTGCCAACAAAGA R: GAGGAGGGGGAACCAGAG	(TC)_29_	269	51	JN625272
LG17 *	F: TCGGGATCATGTAGTTATT R: GTTTACTTTTACCATGCTTCTA	(CA)_12_- (GA)_21_	148–160	59	JN625273
LG22	F: GAATCGGACGAACTTTCT R: TGTAGCCTATCCTACCTCA	(GAA)_7_	150	61	JN625274
LG24	F: GAGAAAGGTGGACTACTGT R: TCGGAGTTCTGATGGGAT	(TTC)_8_	181	59	JN625275
LG25 *	F: CTTCACTTGGACTGGAGC R: TTGAATTTTGTGCTTGGT	(CA)_7_- (GA)_9_	283–289	50	JN625276
LG26 *	F: TTACAAATTGCAAGGAGG R: CCACTTCATCTTCCCTTA	(GA)_13_	108–118	57	JN625277
LG27 *	F: GTGATTTTGCTCTCTCTGTCTCTTT R: AGTGGTTACAATGCTGGT	(TG)_11_	84–90	63	JN625278
LG28	F: AAAGCAGGACAAAGAACAC R: ATTGAGGACGAAGCAGAA	(GA)_14_	194	52	JN625279
LG30	F: ATCGATTATTCACTCACG R: AGTAGTAACCTTGCCAGA	(CT)_29_	162	52	JN625280
LG31	F: CCCAACCAAATGAGATGA R: TTGGCTCTGGTAATAAAGG	(AC)_5_ (AG)_25_	209	54	JN625281
LG33 *	F: GCGGACATCAATTTTAGTACTCTAT R: TGTCTCCAGGACCAAAGG	(GA)_29_	124–133	57	JN625282

* Displayed polymorphisms in *Luculia gratissima; T*_a_: PCR annealing temperature.

**Table 2:** Result of 10 polymorphic microsatellite loci screening in two populations of *Luculia gratissima*.

	Population NMD (*N* = 12)			Population BLG (*N* = 12)		
Locus	*N*_A_	*H*_E_	*H*_O_	*N*_A_	*H*_E_	*H*_O_
LG2	5	0.619	0.333	4	0.608	0.333
LG5	2	0.507	0.000 *	2	0.289	0.333
LG10	3	0.420	0.000 *	4	0.710	0.166 *
LG11	3	0.565	0.166 *	3	0.409	0.083 *
LG13	3	0.619	1.000 *	3	0.561	1.000 *
LG17	4	0.655	0.416	5	0.641	0.333 *
LG25	2	0.344	0.083	3	0.467	0.250
LG26	3	0.565	0.166 *	3	0.554	0.083 *
LG27	2	0.391	0.500	5	0.760	0.333 *
LG33	3	0.681	0.000 *	4	0.739	0.500
**Mean**	3.000	0.536	0.266	3.600	0.573	0.341

*N* = population sample size; *N*_A_: number of alleles revealed; *H*_E_: expected heterozygosity; *H*_O_: observed heterozygosity;

* statistically significant deviation from Hardy-Weinberg equilibrium at *P* < 0.01; Population NMD (Daman, Makwanpur, Narayani: 27°40′N, 85°05′E, 1970 m a.s.l.); Population BLG (Godawari, Lalitpur, Bagmati: 27°40′N, 85°19′E, 1385 m a.s.l.).

## References

[1] Chen WQ (2003). Flora of Yunnan (Chinese Version).

[2] Chen T, Luo XR, Zhu H, Charlotte MT, Friedrich E, Henrik L, Michele F, Christian P, Wu ZY, Raven PH, Hong DY (2011). Rubiaceae. Flora of China.

[3] Murray BG (1989). Heterostyly and Pollen-tube Interactions in *Luculia gratissima* (Rubiaceae). Ann. Bot.

[4] Barrett SCH, Jesson LK, Baker AM (2000). The evolution and function of stylar polymorphisms in flowering plants. Ann. Bot.

[5] Barrett SCH, Shore JS, Franklin-Tong VE (2008). New Insights on Heterostyly: Comparative Biology, Ecology and Genetics. Self-Incompatibility in Flowering Plants.

[6] Zane L, Bargelloni L, Patarnello T (2002). Strategies for microsatellite isolation: A review. Mol. Ecol.

[7] Doyle JJ, Doyle JL (1987). A rapid DNA isolation procedure for small quantities of leaf tissue. Phytochem. Bull.

[8] Clarke KR, Gorley RN (2001). PRIMER (Version 5): User Manual/Tutorial.

[9] Raymond M, Rousset F (1995). GENEPOP (version 1.2): Population genetics software for exact tests and ecumenicism. J. Hered.

